# Supervised Versus Unsupervised Pulmonary Rehabilitation in Patients with Pulmonary Embolism: A Valuable Alternative in COVID Era

**DOI:** 10.3390/jfmk6040098

**Published:** 2021-12-03

**Authors:** Vasileios T. Stavrou, Michalis Griziotis, George D. Vavougios, Dimitrios G. Raptis, Fotini Bardaka, Eleni Karetsi, Athanasios Kyritsis, Zoe Daniil, Konstantinos Tsarouhas, Filippos Triposkiadis, Konstantinos I. Gourgoulianis, Foteini Malli

**Affiliations:** 1 Laboratory of Cardio-Pulmonary Exercise Testing and Pulmonary Rehabilitation, Department of Respiratory Medicine, Faculty of Medicine, University of Thessaly, 41110 Larissa, Greece; vasileiosstavrou@hotmail.com (V.T.S.); mihgrizio@hotmail.co.uk (M.G.); dantevavougios@hotmail.com (G.D.V.); ekaretsi@uth.gr (E.K.); zdaniil@uth.gr (Z.D.); kgourg@uth.gr (K.I.G.); 2 Department of Respiratory Medicine, Faculty of Medicine, University of Thessaly, 41110 Larissa, Greece; raptisdmed@gmail.com (D.G.R.); bardakafotini@yahoo.gr (F.B.); thanoskyrit@hotmail.com (A.K.); 3 Department of Cardiology, University General Hospital of Larissa, 41110 Larissa, Greece; ktsarouhas14@yahoo.gr (K.T.); ftriposkiadis@gmail.com (F.T.); 4 Faculty of Nursing, University of Thessaly, 41500 Larissa, Greece

**Keywords:** exercise, pulmonary embolism, pulmonary rehabilitation

## Abstract

The aim of our study was to assess the effect of 8 weeks of pulmonary rehabilitation (PR) in patients with pulmonary embolism (PE) during unsupervised PR (unSPR_group_) versus supervised PR (SPR_group_) on cardiopulmonary exercise testing (CPET) parameters, sleep quality, quality of life and cardiac biomarkers (NT-pro-BNP). Fourteen patients with PE (unSPR_group_, n = 7, vs. SPR_group_, n = 7) were included in our study (age, 50.7 ± 15.1 years; BMI, 30.0 ± 3.3 kg/m^2^). We recorded anthropometric characteristics and questionnaires (Quality of life (SF-36) and Pittsburg sleep quality index (PSQI)), we performed blood sampling for NT-pro-BNP measurement and underwent CPET until exhausting before and after the PR program. All patients were subjected to transthoracic echocardiography prior to PR. The SPR_group_ differed in mean arterial pressure at rest before and after the PR program (87.6 ± 3.3 vs. 95.0 ± 5.5, respectively, *p* = 0.010). Patients showed increased levels of leg fatigue (rated after CPET) before and after PR (*p* = 0.043 for SPR_group_, *p* = 0.047 for unSPR_group_) while the two groups differed between each other (*p* = 0.006 for post PR score). Both groups showed increased levels in SF-36 scores (general health; *p* = 0.032 for SPR_group_, *p* = 0.010 for unSPR_group_; physical health; *p* = 0.009 for SPR_group_, *p* = 0.022 for unSPR_group_) and reduced levels in PSQI (cannot get to sleep within 30-min; *p* = 0.046 for SPR_group_, *p* = 0.007 for unSPR_group_; keep up enough enthusiasm to get things done; *p* = 0.005 for SPR_group_, *p* = 0.010 for unSPR_group_) following the PR program. The ΝT-pro-BNP was not significantly different before and after PR or between groups. PR may present a safe intervention in patients with PE. The PR results are similar in SPR_group_ and unSPR_group_.

## 1. Introduction

Pulmonary embolism (PE) is an acute and potentially fatal condition in which embolic material, usually a thrombus originating from the deep veins of the legs, blocks the pulmonary circulation resulting in impaired blood flow that may lead to right ventricle dysfunction [1]. PE and deep vein thrombosis are considered to be two manifestations of venous thromboembolism (VTE), which represents the third most common cardiovascular disorder in industrialized countries [2]. PE is difficult to diagnose due to lack of specificity of symptoms and clinical presentation [3]. Patients with history of PE often exhibit functional limitations and decreased quality of life even years after the episode, a condition that is considered as a long-term complication of acute PE and termed “post-PE-syndrome” or “Chronic Thromboembolic Disease” [4].

PE in the setting of COVID-19 is a common complication, frequent in hospitalized patients [5], and is associated with its severity [6]. On the pathophysiological level, the relationship between PE and COVID-19 is bidirectional. Hypercoagulable states and endothelial injury may be induced via virus–host interactions, while subsequent PE may account for persistent hypoxia following the resolution of the acute syndrome [7]. The incidence of PE following COVID-19 varies according to the population studied, the severity of COVID, the thromboprophylaxis dose, the screening protocol for VE, etc. According to a recent meta-analysis, the overall incidence of PE in COVID-19 inpatients is approximately 17%, with increased incidence in patients admitted to ICU (27.9%) versus those hospitalized in wards (7.1%) [8].

The “post-PE-syndrome” is characterized by suboptimal cardiac function but not pulmonary hypertension, altered pulmonary artery flow dynamics, and impaired oxygenation at rest or at exercise, associated with symptoms such as dyspnea, reduced exercise tolerance, or worsening of quality of life, that cannot be explained otherwise [4]. The pathophysiology of the syndrome is poorly understood while its treatment is not specified to measures other than anticoagulation and supportive care. Recent guidelines have outlined that an efficient follow-up strategy after PE should include exercise rehabilitation, although studies addressing the effect of pulmonary rehabilitation programs in these patients are lacking [9]. Pulmonary rehabilitation (PR) includes a supervised program of exercise training and breathing techniques that also addresses issues of health education. PR represents a safe and effective intervention which improves health indicators and quality of life of patients with certain lung diseases such as chronic obstructive pulmonary disease or lung involvement due to other conditions [10]. European Respiratory Society Council and Executive Committee [10] underlies the need to establish specialized rehabilitation programs in order to enhance patient accessibility to this treatment intervention. According to World Health Organization [11], the health indicators related to metabolic profile, physical activity [12], and aerobic and anaerobic capacity are assessed within the cardio-pulmonary exercise testing (CPET). Briefly, CPET is a non-invasive measurement which provides an objective quantitative assessment of metabolic, pulmonary and cardiovascular responses during exercise [13]. Several biomarkers for PE diagnosis, risk stratification and/or risk of recurrence exist but most of them require further validation before being applied in clinical practice. Cardiac troponin T, N-terminal-pro hormone BNP (NT-pro-BNP) and heart-type fatty-acid binding protein, are markers of myocardial strain and injury, which have prognostic value in risk assessment strategies [4].

There is paucity of data concerning the possible role of PR programs in patients with PE. This lack of data extends to unsupervised PR (unSPR_group_), which represents a telemedicine approach that has gained impetus during the COVID-19 pandemic [14]. Telemedicine approaches, including virtual reality applications, have had previous successful implementations in the setting of pulmonary disease rehabilitation. [15]. Home-based unsupervised rehabilitation has been shown to be an effective alternative to formal regimens during the pandemic, ensuring that patients rehabilitation milestones remain on-track following hospitalization [16]. A study from our group has indicated that the efficacy of unsupervised PR in COVID-19 is tangible, and associated with improvements in redox homeostasis, sleep health and anthropometric indices [17]. Considering the overlap between COVID-19 and PE, these studies further demonstrate the rationale and relevance of unsupervised PR in PE.

Currently, there is no study prospectively addressing the efficacy and safety of PR in exercise limitation and quality of life following an episode of PE, despite current guide-lines that suggest that exercise rehabilitation is part of the follow up of these patient group [8]. The effectiveness of different programs of physical activity is not well established but some studies suggest that supervised versus self-selected programs might have similar results [18]. The types of exercise programs in patients with PE have not been addressed in the literature, to this end, we designed this study in order to investigate the effect of 8 weeks of PR in patients with history of an acute episode of PE. Additionally, we aimed to address the results of PR in exercise limitation and quality of life and examine possible differences among patients subjected to supervised versus unsupervised exercise.

## 2. Materials and Methods

### 2.1. Study Population

**Figure 1:**
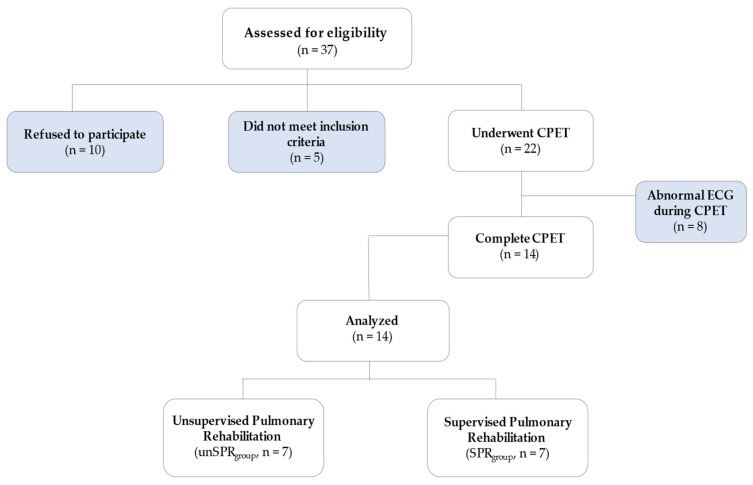
Study flow diagram.

The present research is a pilot study. Patients with a history of PE were prospectively recruited from the PE outpatient clinic (Figure 1) between January 2017 to December 2018. The patients were randomly divided into two groups (using block randomization): unsupervised PR during telerehabilitation (unSPR_group_) and supervised PR (SPR_group_) in Pulmonary Rehabilitation Center (University of Thessaly). Some of these patients were present in a previous study and belong to the Proceedings of the 9th Conference of Biochemistry and Physiology of Exercise [19]. We included patients with PE diagnosis >6 months prior to enrollment and weekly exercise ≤100-min. Exclusion criteria included contraindications to the performance of CPET (i.e., recent acute myocardial infarction (3–5 days), unstable angina, uncontrolled arrhythmia causing hemodynamic instability, acute endocarditis, acute myocarditis or pericarditis, uncontrolled heart failure, lower extremity thrombosis, pregnancy, presence of severe comorbidity that may interfere with the results of the rehabilitation, i.e., COPD). The study was approved by the Institutional Ethics Committee of the corresponding institution. Verbal and written informed consent were obtained from all participants (No. of Ethical Committee: N° 2800, Scientific Council of University Hospital of Larissa).

### 2.2. Procedures

For all patients, we recorded the demographics and characteristics of PE episodes and all subjects underwent echocardiography. Prior to CPET, all participants answered the Pittsburgh Sleep Quality Index (PSQI) [20] and Quality of Life (SF-36) [21] questionnaires while we recorded anthropometric characteristics [22,23,24], pulmonary function parameters (FEV_1_: forced expiratory volume in 1st sec, FVC: forced vital capacity; Master Screen-CPX, VIASYS HealthCare, Hochberg, Germany) [25]. Blood sampling for NT-pro-BNP measurement was performed 30 min before CPET. BNP measurements were performed in complete blood samples with a commercial analyzer (Triage BNP test; BI-OSITE, San Diego, CA, USA). The same procedure was repeated after 8 weeks.

### 2.3. Echocardiography

All patients underwent echocardiographic study within 48 h of the CPET and PR program initiation. Two-dimensional echocardiography was performed, with the subjects resting in a left lateral decubitus position, using a Vivid BT08 (General Electric, Miami, FL, USA). Heart images were obtained in the standard parasternal long-axis and short-axis and apical four-and-two chamber planes. Wall thickness was measured from 2D short-axis views at end-diastole, with the greatest measurement within the left ventricular wall defined as the maximal wall thickness. M-mode echocardiograms derived from 2D images in the parasternal long axis were used for the measurement of end-diastolic and systolic dimensions according to the American Society of Echocardiography [26].

### 2.4. Cardiopulmonary Exercise Testing

CPET was performed on an electronic cycle ergometer (Ergoselect 100, Bitz, Germany) Master Screen-CPX and respiratory and cardiac parameters were recorded (VIASYS HealthCare, Höchberg, Germany). All patients, prior to testing, were familiarized with the test via a 2 min resting stage (1st stage); for a 3 min unloaded cycling as a warm-up (2nd stage); then, after the end of the maximal test (3rd stage), they performed a 5 min unloaded cycling for recovery (4th stage) purposes. In the 3rd stage the ramp work rate increased by 10–15 watts/min until exhaustion was reached. The work rate increment calculated using the Wasserman et al. [27] formula:

Work Rate/min _(ramp)_ = (*V*O_2max_ − *V*O_2unloaded_)/100

*V*O_2max_ = (Height _(cm)_ − Age _(years)_) × 20 (male) or × 14 (female)

*V*O_2unloaded_ = 150 + (6 × Weight _(kg)_)

During testing all patients were instructed to keep a steady speed of 60 ± 5 rpm throughout the four phases of testing. Each trial was terminated when the participant reached symptom-limited maximum exercise, which was confirmed by the presence of respiratory exchange ratio (RER) > 1.10, Heart Rate (HR) ≥ 80% of predicted HR_max_, and/or plateau of oxygen consumption with increasing work load. Moreover, a 12-lead electrocardiogram (ECG) was also employed for HR monitoring, while a pulse oxymeter (MasterScreen, Höchberg, Germany) informed about oxygen saturation (SpO_2_). Blood pressure (cuff manometry, Mac, Japan) and Borg CR10 Scales (Leg Fatigue, Dyspnea) point were recorded every 2 min for all phases.

### 2.5. Pulmonary Rehabilitation Program

The PR program lasted 8 weeks with three sessions per week. The duration of each training session was about 70 min. All sessions were instructed to conduct the PR program either outdoors (walking) or in home (stretching, strength and breathing exercise) without supervision for patients of the unSPR_group_. Patients in the SPR_group_ performed the PR program in the Laboratory of Pulmonary Rehabilitation of the University Hospital of Larissa. Each training session included a warm-up (unSPR_group_ and SPR_group_: 5 min stretching exercises), the first main set (unSPR_group_: 40 min walking at 60% of VO_2_ calculated from heart rate vs. SPR_group_: 40 min intermittent exercise in cycle ergometer (30 s exercise at 70% of VO_2max_ and 30 s resting)), the second main set of training (un-SPR_group_: 10 min (tele)breathing physiotherapy and 10 min multi-joint strength exercises vs. SPR_group_: 10 min respiratory physiotherapy and 10 min multi-joint strength exercises) and a recovery set (unSPR_group_ and SPR_group_: 5 min stretching exercises). The set of exercises was analogue for both groups. Minor differences in the two PR programs exist in order to increase safety for injuries. The unSPR_group_ performed exercise-PR unsupervised but according to instructions of a clinical exercise physiologist (VTS) that supervised the SPR_group_.

Adherence to the program of unSPR_group_ was determined via phone calls per week. Each call focused on whether the patients were able to follow the instructions and perform them, troubleshooting and reporting the physiological parameters.

### 2.6. Statistical Analysis

The Kolmogorov–Smirnov test was utilized to assess normality of distribution of values. A comparison of one group of individuals against themselves (pre-and-post the PR) was performed with the Wilcoxon signed-rank test according to variable distribution. A comparison between the two patient groups was performed with the use Mann–Whitney U-test according to variable distribution. Data are presented as absolute numbers, percentages or mean values and standard deviation (mean ± SD). For all the statistical analyses the statistical package SPSS 15 (SPSS Inc., Chicago, IL, USA) was used. The level of significance was set at *p* < 0.05.

## 3. Results

Out of the 37 individuals who were assessed for eligibility, 14 were included in the study (Figure 1). Table 1 and Table 2 presents demographical, clinical and echocardiography results of the study subjects. Briefly, the SPR_group_ vs. the unSPR_group_ did not differ significantly in terms of age (49.6 ± 15.4 vs. 51.9 ± 16.0, respectively), gender distribution (males 85.71% vs. 71.42%, respectively) and smoking status (smokers: 28.6% vs. 42.9%, respectively). Similarly, SPR_group_ vs. the unSPR_group_ had similar BMI (29.8 ± 3.9 vs. 30.1 ± 2.9, kg/m^2^, respectively), baseline physical activity (61.7 ± 24.7 vs. 70.0 ± 14.1, respectively) (Table 1). The two groups did not differ at baseline in terms of cardiovascular comorbidities, blood pressure measurements and spirometry results (Table 1). Mean Pulmonary Severity Index (PESI) score of the study participants was 52.48 ± 48.23. All patients were hospitalized during the acute period. Mean PESI score was not significantly different between SPR_group_ and unSPR_group_ (103.25 ± 50.90 vs. 100.33 ± 49.13, *p* > 0.05). Ejection fraction was within the normal range in both SPR_group_ and unSPR_group_ (59.2 ± 2.0 vs. 59.4 ± 1.3, respectively) (Table 2). Echocardiographic signs of right heart dysfunction (i.e., end-diastolic right ventricular diameter in four chambers view, right ventricular systolic pressure, right atrial area, tricuspid annular plane systolic excursion) were not significantly different between the two study groups (Table 2).

Respiratory parameters and CPET results, before and after the PR for both groups, are presented in Table 3. The SPR_group_ differed in mean arterial pressure (MAP) at rest before and after the PR program (87.6 ± 3.3 vs. 95.0 ± 5.5, respectively, *p* = 0.010). MAP levels did not differ significantly before and after the PR program in the unSPR_group_ (85.5 ± 8.5 vs. 88.6 ± 9.2, respectively). We observed an increasing trend in P_ET_O_2_ in the SPR_group_ after the PR program vs. at baseline that did not reach statistical significance (115.0 ± 7.0 vs. 108.7 ± 3.9, respectively, *p* = 0.059) (Table 3). Patients showed differences in leg fatigue before and after PR (SPR_group_: 2.6 ± 1.4 vs. 3.6 ± 1.3, respectively, *p* = 0.043 and unSPR_group:_ 1.1 ± 0.7 vs. 1.7 ± 0.8, respectively, *p* = 0.047). Leg fatigue following the PR program was significantly higher in SPR_group_ when compared to unSPR_group_ (3.6 ± 1.3 vs. 1.7 ± 0.8, respectively, *p* = 0.006).

Both patient groups showed statistically significant differences before and after PR in the Quality of Life and Sleep Quality questionnaires. At baseline no differences were observed in both groups for all the subscales of SF-36 (Table 4). After the PR program, we observed that both groups had a higher score in the SF-36 parameters “*physical health*” and “*general health*” versus to their baseline values. In more detail, “*physical health*” increased significantly after the PR program when compared to baseline in the SPR_group_ (92.9 ± 8.1 vs. 71.1 ± 10.7, respectively, *p* = 0.009) and the unSPR_group_ (93.6 ± 6.9 vs. 76.4 ± 15.7, respectively, *p* = 0.022). “*General health*” score increased following PR vs. before in the SPR_group_ (81.4 ± 16.8 vs. 63.6 ± 9.9, respectively, *p* = 0.032) and the unSPR_group_ (72.1 ± 8.6 vs. 51.4 ± 15.7, respectively, *p* = 0.010). The others parameters of SF-36 were not different before and after PR period (Table 4).

Sleep quality as assessed by PSQI showed differences in both groups before and after PR in parameter “*cannot get to sleep within 30 min*”, (Table 4) and “*keep up enough enthusiasm to get things done*” (Table 4). The PSQI score decreased after PR vs. at baseline (unSPR_group_: 5.7 ± 1.4 vs. 3.9 ± 1.8, respectively, *p* = 0.035; SPR_group_: 6.6 ± 1.8 vs. 4.1 ± 1.8, respectively, *p* = 0.026) compared to the period before PR. The other parameters of PSQI were not different before and after PR period but presented an increasing trend in both groups that did not reach statistical significance.

**Figure 2:**
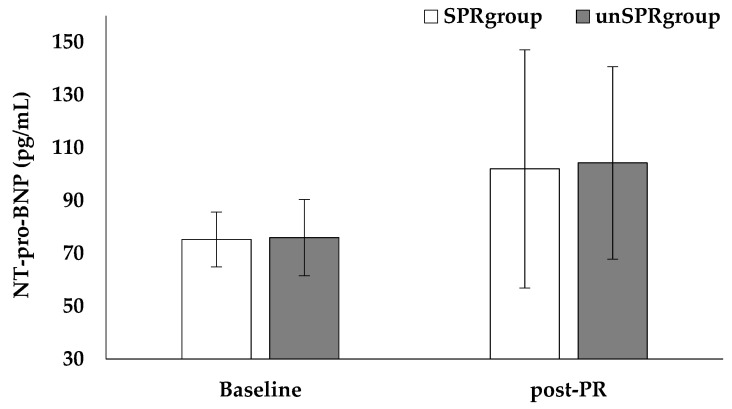
NT-pro-BNP results between groups before and after pulmonary rehabilitation. The bars correspond to the mean value and the outliers correspond to standard deviation.

The ΝT-pro-BNP levels were not significantly different before and after PR (SPR_group_: 75.3 ± 10.4 vs. 102.0 ± 45.1 pg/mL, respectively, *p* = 0.147; unSPR_group_: 76.0 ± 14.4 vs. 104.3 ± 36.5 pg/mL, respectively, *p* = 0.116, Figure 2) or between groups (Baseline, SPR_group_: 75.3 ± 10.4 vs. unSPR_group_: 76.0 ± 14.4, pg/mL, *p* = 0.917; post-PR, SPR_group_: 102.0 ± 45.1 vs. unSPR_group_: 104.3 ± 36.5, pg/mL, *p* = 0.919, Figure 2).

## 4. Discussion

The aim of this pilot study was to investigate the effect of 8 weeks of PR in patients with PE. Patients underwent PR program either by SPR_group_ in a Pulmonary Rehabilitation Center or by unSPR_group_ during telerehabilitation and groups were compared to each other. We observed differences in MAP at rest before and after the PR program in the SPR_group_ while both groups showed differences in leg fatigue before and after PR as well as compared to each other. Importantly, the two group of patients showed differences in parameters of quality of life and sleep quality before and after the PR. We did not observe major differences when SPR_group_ was compared to unSPR_group_ with the exception of reduced leg fatigue reported by the SPR_group_ and the parameter “*keep up enough enthusiasm to get things done*” in PSQI which was in favor of the unSPR_group_. However, due to the small number of patients included, no definite conclusions can be drawn concerning differences in the effectiveness of each approach.

### 4.1. Cardiopulmonary Exercise Testing

CPET provides an objective and quantitative measure of metabolic, pulmonary and cardiovascular responses during exercise which is both non-invasive and safe and can serve as an independent predictor of long-term outcomes [9]. Previous studies have documented reductions in *V*O_2max_ at various single time points following the PE event [28]. Kahn et al. [29] observed persistent exercise limitations 1-year after PE. Our results showed significant differences in MAP (at rest) in the SPR_group_ before and after PR while both groups showed differences in leg fatigue pre-and-post PR. Additionally, we demonstrated differences in leg fatigue between groups (SPR_group_ versus unSPR_group_). CPET may be limited by leg fatigue that may underlie muscle weakness and leg effort [30]. Most patients tolerate only minimal leg discomfort before stopping the measurement while average healthy individuals may tolerate a greater degree of discomfort, suggesting the subjectiveness of the intensity of leg effort [30]. Leg fatigue may be associated with muscle metabolism impairment and probably increased peripheral oxygen uptake [31]. These findings are of significant clinical relevance and represent the standard of care for the management of patients with cardiovascular diseases such as pulmonary hypertension [32]. According to Kwan et al. [33], the use of exercise training programs of cardiac rehabilitation may benefit patients with a history of acute PE in a manner similar to that of patients suffering acute coronary syndromes.

### 4.2. Quality of Life and Psychological Aspects

Previous studies have shown that PR may have beneficial effects in patients with cardiovascular diseases, in terms of improved functional capacity and quality of life [34]. Quality of life has become an important outcome aspect of medical care. Patients with PE may have reduced chronic functional capacity for many years after the event and that may be the main determinant of impaired quality of life [34]. Our results showed difference before and after PR in quality of life, as assessed by the SF-36 questionnaire, in parameters such as “*general health*” and “*physical health*”. Reduced functional capacity may relate to persistent dyspnea, while physical activity and exertion were the most common behavior changes in patients. In our study, patients with PE during rehabilitation performed combination exercise and respiratory physiotherapy. This combination may relate to behavior change in patients following PR. Although not statistically significant, we observed a trend for improvement in exercise capacity which may be attributed to improved muscle function and desensitization to dyspnea. Desensitization to dyspnea is often considered a mechanism to explain benefit in the rehabilitation of patients with respiratory diseases and these altered perceptions of dyspnea even without associated physiological changes [34]. It should be noted that the interventions via PR/uns-PR also affect several psychological components. PR for chronic lung diseases has been shown to reduce anxiety and depression in these patients [35]. Telemedicine approaches, either as simple as a follow-up via phone [36] or as intricate as virtual reality [15] have also been shown to confer the same beneficial effect in both anxiety and depression experienced by these patients. A limitation in our study, however, is that we did not specifically assess these parameters, and therefore cannot comment on how rehabilitation may have affected them in this patient population.

### 4.3. Sleep Quality

Sleep disorder breathing and PE are a major health issue in industrialized countries. PE patients have a 2–4 times greater risk of suffering from moderate and severe obstructive sleep apnea syndrome (OSAS) [37]. Previous studies suggest that intermittent hypoxia and fragmentation of sleep may result in blood hypercoagulability, endothelial dysfunction and venous stasis. According to García-Ortega et al. [38], patients with acute PE have increased risk of coexisting moderate and severe OSAS when compared with controls. A polysomnography study showed a lower degree of oxygen saturation in PE patients and higher risk of PE in patients with isolated OSAS while the high hypoxic burden may be related to PE prevalence [39]. Patients with acute PE are at an increased risk of coexisting sleep disorders [38]. Our results showed a difference in sleep quality according to the PSQI questionnaire, in parameters such as “*cannot get to sleep within 30-min*” and “*keep up enough enthusiasm to get things done*” following the PR program. According to Stavrou et al. [40,41], the exercise, in patients with sleep disorders, may reduce the apnea-hypopnea index and improve the sleep quality while the daily physical activity may have a protective role in disease progression.

### 4.4. Implication of Rehabilitation Program

Data concerning rehabilitation after an acute episode of PE are sparse, while the literature lacks data concerning pulmonary rehabilitation program following PE recovery. Many patients have persistent symptoms months after an acute episode of PE and some of those suffer from post-PE syndrome, characterized by exercise limitation and suboptimal right heart function [4]. These patients may benefit the most from PR programs; however, data on this population are lacking. Rehabilitation program may be safe in the PE population in terms of death and serious events [42]. Exercise training after VTE showed differences in *V*O_2max_ in previous studies with 3-month intervention duration [43]. To our knowledge, this is the first study addressing the effectiveness and feasibility of an unsupervised PR in patients with a PE. The results of our study may help to establish a specialized rehabilitation program with potential important benefits and provide data on the safety, which seems to be a valuable alternative during the pandemic, of exercise programs in pulmonary embolism patient. Moreover, PR could be a highly valuable tool for promoting exercise and symptom recovery following pulmonary embolism and a novel approach concerning the treatment of persistently symptomatic patients with PE may arise.

### 4.5. Biomarkers

Several biomarkers have been implicated in the diagnosis and risk stratification of PE. Systemic biomarkers of myocardial injury and ventricular dysfunction have been extensively used in everyday clinical practice for the initial risk assessment of patients with acute PE [8]. High levels of BNP may help in the identification of subjects with PE and higher risk of in-hospital complications and death [44]. Additionally, NT-pro-BNP has emerged as a potential biomarker of early diagnosis of Chronic Thromboembolic Pulmonary Hypertension [45]. However, little is known about NT-pro-BNP levels in patients following an acute PE episode, as well as its possible association with exercise limitation. We did not observe differences in NT-pro-BNP levels among patients before and after PR program. We acknowledge that the strength of our results is limited due to the small number of patients included and therefore we suggest that further studies are warranted in order to exert any definite conclusions.

## 5. Conclusions

In conclusion, pulmonary rehabilitation has beneficial effects on quality of life and sleep quality in patients with pulmonary embolism. It also shows an improving tendency of indicators related to physical capacity. There were no major differences between SPR_group_ and unSPR_group_ (except reduced leg fatigue in the SPR_group_ and improvement in PSQI parameter “*keep up enough enthusiasm to get things done*” in the unSPR_group_). Uns-PR may be a feasible alternative to supervised regimens in the pandemic era, considering its relevance to PE and the increased prevalence of PE due to COVID-19. A larger trial is needed to extend these observations and provide evidences for the long-term effects of pulmonary rehabilitation and confirm the findings.

## Figures and Tables

**Table 1:** Demographical, clinical and spirometry results of the study population.

	SPR_group_	UnSPR_group_	*p* Value
Age, years	49.6 ± 15.4	51.9 ± 16.0	0.790
Gender, M/F	6/1	5/2	0.552
Smokers, %	28.6	42.9	0.611
Body mass index, kg/m^2^	29.8 ± 3.9	30.1 ± 2.9	0.884
Body surface area, m^2^	2.0 ± 0.5	2.2 ± 0.3	0.537
Lean body mass, %	60.9 ± 9.0	63.6 ± 6.3	0.531
Total body water, L	44.2 ± 8.9	44.7 ± 7.0	0.904
Alcohol drinking, ml/month	85.0 ± 13.7	83.3 ± 14.4	0.875
Physical Activity, min/week	61.7 ± 24.7	70.0 ± 14.1	0.703
Systolic blood pressure, mmHg	113.6 ± 13.8	117.1 ± 9.9	0.588
Diastolic blood pressure, mmHg	71.4 ± 6.3	72.9 ± 2.7	0.589
Prevalence of CVD and diabetes, %	42.9	57.1	0.626
Prior VTE event, Y/N	1/6	1/6	1.000
Provoked event, Y/N	3/4	6/1	0.109
Under anticoagulant therapy, Y/N	6/1	4/3	0.271
MRC dyspnea scale, 0/I/II	3/4/0	2/4/1	0.690
FEV_1_, % of predicted	101.0 ± 8.0	96.9 ± 10.2	0.434
FVC, % of predicted	96.8 ± 7.7	90.9 ± 11.6	0.280

Abbreviations: CDV, cardiovascular disease; FEV_1_, forced expiratory volume in 1st sec; FVC, forced vital capacity; M/F, male/female; VTE, venous thromboembolism event; Y/N, yes/no.

**Table 2:** Echocardiographic characteristics of the study population.

	SPR_group_	UnSPR_group_	*p* Value
Ejection fraction, %	59.2 ± 2.0	59.4 ± 1.3	0.832
End-diastolic RV diameter (4CH), cm	3.6 ± 0.1	3.6 ± 0.5	0.763
RVSP, mmHg	23.3 ± 4.1	24.2 ± 5.8	0.777
RA area, cm^2^	15.3 ± 2.5	15.2 ± 3.1	0.952
TAPSE, mm	20.5 ± 7.8	22.3 ± 4.3	0.626

Abbreviations: RA, right atrial; RV, right ventricle; RVS, right ventricular systolic pressure; TAPSE, tricuspid annular plane systolic excursion.

**Table 3:** Pulmonary function parameters and cardiopulmonary exercise testing results between groups before and after the pulmonary rehabilitation program. Continuous variables are presented as mean ± standard deviation.

	SPR_group_			UnSPR_group_			*p* Value between Groups	
	Baseline	Post-PR	*p* Value	Baseline	Post-PR	*p* Value	PR_pre_	PR_post_
Resting								
*V*O_2_, mL/min	330.6 ± 91.6	349.0 ± 120.8	0.735	336.3 ± 84.5	312.0 ± 53.9	0.533	0.905	0.473
*V*CO_2_, mL/min	257.1 ± 67.1	240.6 ± 101.8	0.095	267.9 ± 102.7	236.1 ± 32.9	0.452	0.821	0.095
P_ET_CO_2_, mmHg	29.9 ± 3.7	29.5 ± 2.4	0.832	27.2 ± 4.1	27.1 ± 3.6	0.952	0.227	0.159
P_ET_O_2_, mmHg	110.0 ± 5.1	114.0 ± 4.2	0.132	113.8 ± 7.7	112.5 ± 5.4	0.736	0.305	0.568
HR, bpm	81.0 ± 18.2	82.6 ± 18.2	0.875	78.7 ± 10.1	73.9 ± 9.6	0.374	0.776	0.285
MAP, mmHg	87.6 ± 3.3	95.0 ± 5.5	0.010	85.5 ± 8.5	88.6 ± 9.2	0.516	0.549	0.133
Maximal effort								
*V*O_2_, mL/min	1559.1 ± 372.8	1579.3 ± 430.7	0.927	1946.0 ± 640.2	1896.1 ± 390.0	0.863	0.192	0.175
*V*CO_2_, mL/min	1536.6 ± 440.8	1525.5 ± 497.6	0.966	1954.1 ± 672.1	1791.0 ± 453.9	0.604	0.194	0.318
P_ET_CO_2_, mmHg	37.5 ± 4.1	36.7 ± 3.5	0.710.	35.5 ± 4.6	35.6 ± 4.4	0.970	0.416	0.615
P_ET_O_2_, mmHg	108.7 ± 3.9	115.0 ± 7.0	0.059	112.8 ± 3.8	115.9 ± 4.8	0.199	0.079	0.788
V_E_/MVV, %	44.1 ± 10.8	48.1 ± 10.5	0.494	53.3 ± 6.3	53.5 ± 12.7	0.977	0.076	0.408
V_E_/*V*CO_2_	28.6 ± 3.0	27.8 ± 3.0	0.279	28.5 ± 2.5	28.2 ± 3.3	0.786	0.357	0.679
HR, bpm	133.3 ± 18.6	133.0 ± 14.2	0.975	149.0 ± 13.3	138.4 ± 12.9	0.158	0.094	0.470
MAP, mmHg	119.5 ± 18.8	117.2 ± 10.6	0.786	123.3 ± 12.5	121.4 ± 9.4	0.757	0.660	0.438
Leg fatigue, Borg Scale	2.6 ± 1.4	3.6 ± 1.3	0.043	1.1 ± 0.7	1.7 ± 0.8	0.047	0.062	0.006
Dyspnea, Borg Scale	1.4 ± 1.0	2.0 ± 1.2	0.337	0.9 ± 1.1	1.3 ± 0.8	0.403	0.317	0.196

Abbreviations: HR, heart rate; MAP, mean arterial pressure; MVV, maximum voluntary volume; P_ET_CO_2_, end-tidal carbon dioxide pressure; P_ET_O_2_, end-tidal oxygen pressure; *V*CO_2_, carbon dioxide output; V_E_, minute ventilation; *V*O_2_, oxygen uptake.

**Table 4:** Quality of life and sleep quality results between groups before and after pulmonary rehabilitation. Continuous variables are presented as mean ± standard deviation.

		SPR_group_			UnSPR_group_			*p* Value between Groups	
		Baseline	Post-PR	*p* Value	Baseline	Post-PR	*p* Value	Baseline	Post-PR
Quality of life (SF-36)	Physical Health	71.1 ± 10.7	92.9 ± 8.1	0.009	76.4 ± 15.7	93.6 ± 6.9	0.022	0.923	0.862
	Physical Functioning	78.6 ± 26.7	92.9 ± 18.9	0.271	75.0 ± 25.0	76.4 ± 25.3	0.917	0.801	0.194
	Body Pain	82.5 ± 18.0	87.5 ± 13.4	0.567	80.0 ± 17.0	83.9 ± 13.8	0.643	0.794	0.631
	General Health	63.6 ± 9.9	81.4 ± 16.8	0.032	51.4 ± 15.7	72.1 ± 8.6	0.010	0.109	0.217
	Vitality	67.9 ± 16.0	77.1 ± 13.2	0.260	60.7 ± 13.7	68.6 ± 8.0	0.214	0.387	0.167
	Social Role Functioning	75.0 ± 20.4	92.9 ± 12.2	0.070	85.7 ± 18.3	85.4 ± 14.1	0.968	0.321	0.308
	Emotional Role Functioning	85.7 ± 26.2	98.6 ± 2.4	0.175	85.7 ± 26.2	90.5 ± 16.2	0.690	0.989	0.147
	Mental Health	73.6 ± 24.0	78.3 ± 19.6	0.635	77.1 ± 17.5	81.1 ± 12.2	0.629	0.692	0.749
Sleep quality (PSQI)	Cannot get to sleep within 30 min	2.9 ± 0.4	1.8 ± 0.5	0.046	2.6 ± 0.5	2.0 ± 0.1	0.007	0.779	0.025
	Wake up in the middle of the night or early morning	1.4 ± 0.3	1.4 ± 0.4	0.968	1.3 ± 0.1	1.3 ± 0.2	0.878	0.613	0.694
	Have to get up to use the bathroom	1.1 ± 0.1	1.0 ± 0.5	0.317	1.1 ± 0.4	0.9 ± 0.7	0.986	0.955	0.613
	Cannot breathe comfortably	1.2 ± 0.3	1.1 ± 0.4	0.867	1.1 ± 0.3	1.1 ± 0.6	0.831	0.986	0.978
	Cough or snore loudly	1.0 ± 0.2	0.9 ± 0.6	0.317	0.9 ± 0.3	0.8 ± 0.4	0.326	0.694	0.281
	Feel too cold	1.1 ± 0.3	1.0 ± 0.6	0.325	1.0 ± 0.4	0.9 ± 0.7	0.679	0.732	0.796
	Feel too hot	1.0 ± 0.1	1.0 ± 0.2	0.371	1.1 ± 0.2	0.9 ± 0.3	0.317	0.698	0.789
	Had bad dreams	1.2 ± 0.2	1.1 ± 0.1	0.157	1.1 ± 0.3	1.0 ± 0.2	0.175	0.121	0.779
	Have pain	0.8 ± 0.1	0.7 ± 0.4	0.152	0.8 ± 0.2	0.6 ± 0.8	0.336	0.956	0.232
	…taken medicine to help you sleep	-	-	/	-	-	/	/	/
	…trouble staying awake (driving, eating meals, or social activity)	0.6 ± 0.3	0.6 ± 0.5	0.317	0.5 ± 0.1	0.5 ± 0.6	0.307	0.463	0.397
	…keep up enough enthusiasm to get things done?	1.9 ± 0.4	1.0 ± 0.2	0.005	2.5 ± 0.5	1.3 ± 0.4	0.010	0.779	0.029
	…sleep quality overall	0.3 ± 0.1	0.3 ± 0.2	0.157	0.2 ± 0.2	0.1 ± 0.4	0.317	0.294	0.281

## Data Availability

The data presented in this study are available on request from the corresponding author.
